# The role of the Mod-MPI in identifying cardiac dysfunction in FGR fetuses stratified by umbilical artery flow abnormalities

**DOI:** 10.1186/s12884-025-08120-y

**Published:** 2025-09-18

**Authors:** Tuğçe Arslanoğlu, Kübra Kurt Bilirer, Sezin Uludağ, Hale Çetin Arslan, Merih Çetinkaya, Banu Kılıçoğlu Dane, İbrahim Polat

**Affiliations:** 1 Obstetrics and Gynecology Clinic, Department of Perinatology, University of Health Sciences, Kanuni Sultan Süleyman Training and Research Hospital, Istanbul, Turkey; 2 Obstetrics and Gynecology Clinic, Department of Perinatology, University of Health Sciences, Başakşehir Çam and Sakura City Hospital, Istanbul, Turkey; 3 Obstetrics and Gynecology Clinic, University of Health Sciences, Kanuni Sultan Süleyman Training and Research Hospital, Istanbul, Turkey; 4 Department of Pediatrics, Division of Neonatology, University of Health Sciences, Başakşehir Çam and Sakura City Hospital, Istanbul, Turkey

**Keywords:** FGR, AEDF, MPI, Cardiac dysfunction, Fetal echocardiography

## Abstract

**Background:**

Fetal growth restriction (FGR) is a major cause of perinatal morbidity and mortality. In patients with absent end-diastolic flow (AEDF) in the umbilical artery, placental insufficiency is typically severe, and adverse neonatal outcomes are common. The modified myocardial performance index (Mod-MPI) provides a noninvasive assessment of global fetal cardiac function; however, its relationship with Doppler findings and perinatal outcomes in patients with FGR remains under investigation.

**Methods:**

This prospective observational study included 217 singleton pregnancies between 24 + 0 and 36 + 0 weeks of gestation. Among these, 103 fetuses were diagnosed with FGR and subdivided on the basis of the presence (*n* = 47) or absence (*n* = 56) of AEDF. The control group included 114 gestational age-matched fetuses with normal growth and Doppler findings. Left ventricular Mod-MPI and cardiac time intervals were measured via a standardized pulsed-wave Doppler technique on the basis of valvular motion timing. The mitral inflow E- and A-wave velocities were also recorded. Perinatal outcomes such as gestational age at delivery, birth weight, 5-minute Apgar score, and NICU admission were compared.

**Results:**

Although the mean Mod-MPI values were not significantly different between the groups (*p* = 0.38), AEDF-positive fetuses had shorter ejection times and significantly lower mitral E and A velocities (*p* < 0.001). These findings indicate impaired diastolic function. Compared with other groups, AEDF-positive fetuses were delivered earlier, had lower birth weights, and had higher NICU admission rates (*p* < 0.01).

**Conclusions:**

In fetuses with FGR, the presence of AEDF is associated with early signs of cardiac dysfunction and poor perinatal outcomes. While the mean Mod-MPI may not differ markedly, its components reflect significant hemodynamic compromise. Mod-MPI may be a useful adjunct for monitoring fetal well-being in cases of severe placental insufficiency.

## Introduction

Fetal growth restriction (FGR) is a significant obstetric complication characterized by the failure of the fetus to reach its genetic growth potential, affecting approximately 5–10% of all pregnancies. It is associated with an increased risk of perinatal morbidity and mortality. FGR may result from various etiologies, including uteroplacental insufficiency, chromosomal abnormalities, maternal diseases, and intrauterine infections [1]. These fetuses are at heightened risk for prematurity, perinatal asphyxia, neurodevelopmental impairments, and long-term cardiovascular disease. Therefore, early diagnosis and appropriate management of FGR are critically important for improving fetal and neonatal outcomes [2].

In fetuses with FGR, the cardiovascular system is exposed to increased afterload and reduced preload due to placental insufficiency, leading to early structural and functional alterations in the fetal heart [3]. The myocardial performance index (MPI) has been defined as a noninvasive indicator of fetal cardiac function and provides a combined assessment of both systolic and diastolic function. It offers reliable information on fetal cardiac performance independent of factors such as fetal heart rate, ventricular geometry, and fetal cardiac size [4, 5].

Ultrasonographic Doppler assessments commonly used in the diagnosis of fetal growth restriction (FGR) include evaluations of the umbilical artery (UA), middle cerebral artery (MCA), and ductus venosus (DV) blood flow, providing insight into both uteroplacental and fetal hemodynamics. Among these, absent end-diastolic flow (AEDF) in the umbilical artery represents a critical finding, indicating severely impaired uteroplacental perfusion and a heightened risk of fetal hypoxia during the perinatal period [6]. The presence of AEDF has been directly associated with adverse perinatal outcomes, including fetal acidosis, low Apgar scores, the need for emergency delivery, and admission to neonatal intensive care units [7].

Although conventional Doppler parameters are frequently utilized in the evaluation of FGR, they may not adequately reflect the degree of hemodynamic compromise in all cases and often have limited diagnostic sensitivity. Fetal cardiac dysfunction may occur even in the presence of normal Doppler findings. Therefore, beyond arterial flow assessment, the evaluation of fetal myocardial function is crucial [8]. The aim of this study was to evaluate the modified myocardial performance index (Mod-MPI) in fetuses diagnosed with FGR and to investigate its relationship with fetal hemodynamic alterations—particularly those associated with severe Doppler findings such as AEDF—as well as its correlation with perinatal outcomes.

## Materials and methods

This study was designed as a prospective, cross-sectional, observational investigation conducted at the Perinatology Department of Başakşehir Çam and Sakura City Hospital between February 2023 and April 2024. Within this period, all singleton pregnancies that were diagnosed with fetal growth restriction (FGR) and admitted to our clinic between 24 + 0 and 36 + 0 weeks of gestation were included. On the basis of umbilical artery Doppler findings, these patients were divided into two subgroups: those with absent end-diastolic flow [AEDF (+)] and those without [AEDF (–)].

Gestational age-matched pregnancies with normal biometric measurements and Doppler parameters during the same period served as the control group. A total of 217 singleton pregnancies were evaluated, including 103 in the FGR group and 114 in the control group. A priori sample size calculation was performed using G*Power 3.1 software (effect size f = 0.25, α = 0.05, power = 0.80, three groups), which indicated that at least 159 cases were required; the final study population included 217 fetuses, thus exceeding the calculated sample size. On the basis of fetal growth and umbilical artery Doppler findings, the participants were categorized into three groups:

- Group 1 (FGR + AEDF): Pregnancies with an estimated fetal weight (EFW) below the 10th percentile and no end-diastolic flow in the umbilical artery.
- Group 2 (FGR–AEDF): Pregnant patients diagnosed with FGR but without AEDF in the umbilical artery.
- Group 3 (Control): Pregnant patients whose biometric measurements were appropriate for gestational age and whose Doppler parameters were normal.

The diagnosis of fetal growth restriction (FGR) was established according to the Delphi consensus criteria published in 2020 by the International Society of Ultrasound in Obstetrics and Gynecology (ISUOG) [9]. According to these criteria, the diagnosis of early-onset FGR (≤ 32 weeks) requires an estimated fetal weight (EFW) below the 3rd percentile or an EFW below the 10th percentile accompanied by abnormal Doppler findings (UA PI > 95th percentile, AEDF, DV PI > 95th percentile, or pathological a-wave). For late-onset FGR (> 32 weeks), the diagnosis is based on an EFW below the 3rd percentile or the presence of hemodynamic disturbances indicating cerebral redistribution, such as CPR < 1 or MCA PI < 5th percentile, in conjunction with an EFW below the 10th percentile.

The exclusion criteria included multiple pregnancies, fetuses with structural or chromosomal anomalies, suspected intrauterine infections, maternal systemic diseases (e.g., chronic hypertension, diabetes, or connective tissue disorders), and cases in which technically reliable Mod-MPI measurements could not be obtained. FGR fetuses with reversed end-diastolic flow (REDF) in the umbilical artery were also excluded, as REDF represents the most advanced stage of placental insufficiency and is associated with profound cardiovascular compromise. Including such cases could disproportionately shift Mod-MPI distributions and obscure differences between AEDF (+) and AEDF (–) subgroups, thereby reducing intragroup homogeneity. 

### Ultrasonography and Doppler examination

All ultrasonographic and Doppler evaluations were performed by the same experienced perinatologist via the Fujifilm Arietta 850 (Fujifilm Healthcare, Tokyo, Japan) ultrasound system. A convex abdominal transducer (LISENDO™ C251) was used for imaging. Fetal biometric measurements (BPD, HC, AC, FL) were calculated via the Hadlock formula, and the estimated fetal weight (EFW) was determined accordingly.

During Doppler assessments, pulsatility index (PI) values were measured for the umbilical artery (UA), middle cerebral artery (MCA), and ductus venosus (DV), and the cerebroplacental ratio (CPR = MCA PI/UA PI) was calculated. Throughout all the measurements, care was taken to align the Doppler sampling line (cursor) as parallel as possible to the direction of blood flow, and the insonation angle was optimized to remain within 0–15°. The sweep speed was set at 5 cm/s to ensure optimal temporal resolution for cardiac cycle analysis.

Abnormal Doppler findings were defined on the basis of the absolute (numerical) values of each parameter, according to the following criteria:

- UA PI > 95th percentile or the presence of AEDF.
- MCA PI < 5th percentile,
- DV PI > 95th percentile or absent/reversed a wave,
- CPR < 5th percentile or CPR < 1.

Myocardial Performance Index (MPI) Measurement.

Fetal cardiac function was assessed via the modified myocardial performance index (Mod-MPI). The measurements were obtained through pulsed-wave Doppler recordings of the opening and closing times of the mitral and aortic valves on the basis of the apical four-chamber view. The MPI was calculated via the following formula:

$$\mathrm{Mod}-\mathrm{MPI}=\left(\mathrm{ICT}+\mathrm{IRT}\right)/\mathrm{ET}$$

ICT: Isovolumetric contraction time.

IRT: Isovolumetric relaxation time.

ET: Ejection time.

In addition, the peak velocities of early diastolic filling (E wave) and atrial contraction (A wave) across the mitral valve were measured via pulsed-wave Doppler. The sample volume was positioned at the tips of the mitral valve leaflets and oriented perpendicular to the left ventricular inflow. Valve click alignment was ensured by synchronizing the Doppler tracing with atrioventricular and semilunar valve closure–opening events, while cursor tilt error was minimized by obtaining the recordings from the true apical four-chamber view along the midline insonation plane.

**Fig. 1:**
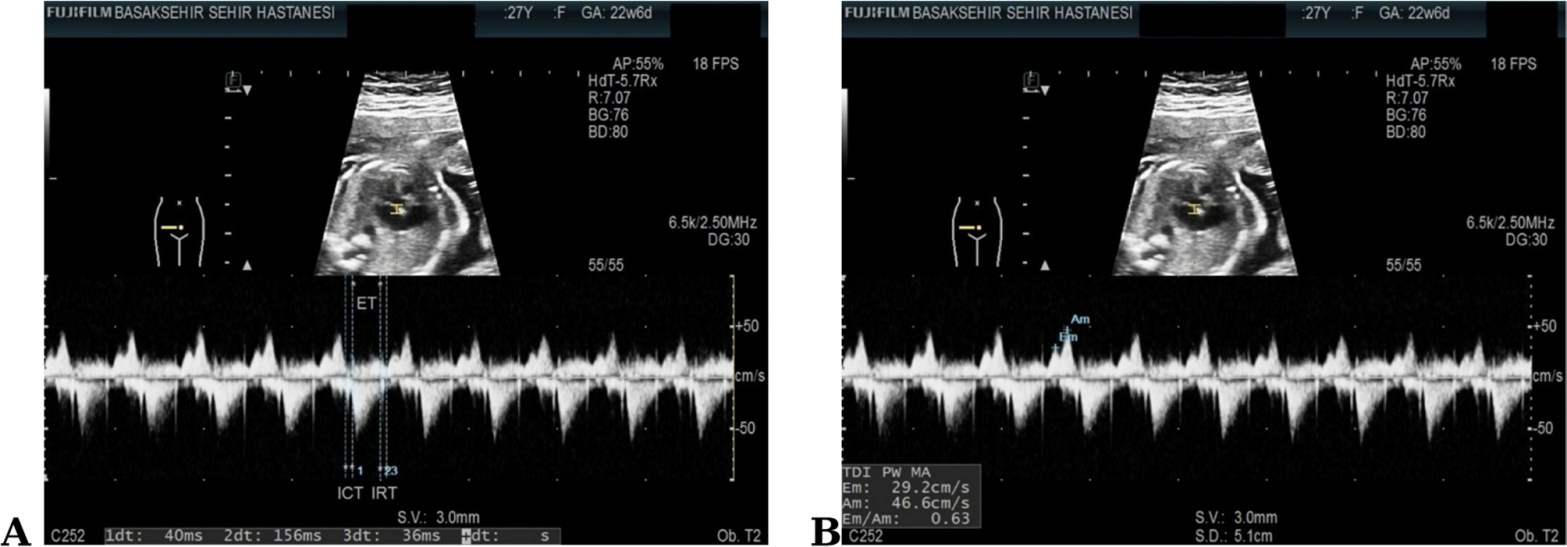
**A** Measurement of ICT, IRT, and ET. **B** Measurement of MV-E PV and MV-A PV

For each case, three consecutive Mod-MPI and E–A wave measurements were obtained, and the mean value was used for analysis. After determining the peak early diastolic (E wave) and atrial contraction (A wave) mitral inflow velocities, the E/A ratio was calculated as an additional index of left ventricular diastolic function. All measurements were performed by the same investigator in accordance with the methodology described by Hernandez-Andrade et al. [10], with the fetal heart rate maintained between 120 and 160 bpm. Representative measurements from our study are presented in Fig. 1.

All patients were followed up until delivery. Perinatal data, including gestational age at delivery, mode of delivery, birth weight, 5-minute Apgar score, and the need for neonatal intensive care unit (NICU) admission, were recorded.

### Ethical approval

This study was approved by the Clinical Research Ethics Committee of Başakşehir Çam and Sakura City Hospital (Ethics Committee Protocol No: 2023- 11). All procedures were conducted in accordance with the ethical standards of the 1964 Declaration of Helsinki and its later amendments. All the volunteers who participated in this prospective study were informed about the scope of the research and provided written informed consent. All the data related to the participants were handled in accordance with confidentiality principles, and no personally identifiable information was shared at any stage or on any platform.

### Statistical analysis

Statistical analyses were performed via NCSS (Number Cruncher Statistical System) 2007 software (Kaysville, Utah, USA). For the evaluation of the study data, descriptive statistical methods (means, standard deviations, medians, frequencies, ratios, minimums, and maximums) were used, and the distributions of the variables were assessed via the Shapiro‒Wilk test. For the comparison of more than two groups of quantitative variables, the Kruskal‒Wallis test was used, whereas the Mann‒Whitney U test was applied for comparisons between two groups. Relationships between qualitative variables were evaluated via the chi-square test. Statistical significance was considered at p<0.01 and p<0.05.

## Results

This study aimed to evaluate the modified left ventricular myocardial performance index (Mod-MPI) in fetuses diagnosed with fetal growth restriction (FGR), grouped according to umbilical artery Doppler findings. A total of 217 pregnancies were examined, including 103 fetuses in the FGR group and 114 fetuses in the normal pregnancy control group. The 103 fetuses in the FGR group were further divided into two subgroups on the basis of the presence or absence of absent end-diastolic flow (AEDF) in the umbilical artery: AEDF (+) (*n* = 47) and AEDF (–) (*n* = 56).

Compared with the control group, the FGR group had significantly lower gestational age at examination, gestational age at delivery, and birth weight, as well as higher maternal body mass index (all *p* < 0.001). Umbilical artery pulsatility index and uterine artery pulsatility index were significantly higher in the FGR group (both *p* < 0.001), while cerebroplacental ratio and middle cerebral artery pulsatility index were significantly lower (both *p* < 0.001). Mitral E and A velocities were also significantly reduced in the FGR group (both *p* < 0.001). The mean E/A ratio, however, was similar between the two groups (0.77 ± 0.12 vs. 0.74 ± 0.09, *p* = 0.250) (Table 1).

**Table 1:** Comparison of measurements between the FGR (Fetal growth Restriction) and control groups

		*n*	Ort ± Ss	Min–Max (Median)	*p*
*Age(years)*	***FGR***	103	27.89 ± 6.02	18–44 (28)	0.109
	***Control***	114	29.16 ± 6.32	19–44 (29)	
*Gravida*	***FGR***	103	2.03 ± 1.4	1–7 (1)	0.009**
	***Control***	114	2.37 ± 1.39	1–6 (2)	
*Parity*	***FGR***	103	0.7 ± 0.94	0–5 (0)	0.024*
	***Control***	114	0.95 ± 0.95	0–3 (1)	
*Abort*	***FGR***	103	0.32 ± 0.74	0–5 (0)	0.436
	***Control***	114	0.37 ± 0.67	0–2 (0)	
*Gestational Week (weeks)*	***FGR***	103	30.71 ± 3.66	23–36 (32)	0.042*
	***Control***	114	31.74 ± 2.95	27–36 (33)	
*Birth Week (week)*	***FGR***	103	34.24 ± 3.38	25–41 (35)	0.001**
	***Control***	114	36.26 ± 1.98	34–41 (36)	
	***Control***	114	4.53 ± 3.3	0–12 (4)	
*Body Mass Index (kg/m2)*	***FGR***	103	28.45 ± 3.6	21.05–40.79 (28.16)	0.001**
	***Control***	114	26.46 ± 3.04	21.91–33.5 (27.24)	
*ICT (ms)*	***FGR***	103	38.68 ± 7.61	24–60 (40)	0.046*
	***Control***	114	40.74 ± 7.75	24–64 (40)	
*IRT (ms)*	***FGR***	103	44.17 ± 7	32–76 (44)	0.053
	***Control***	114	42.11 ± 6.03	32–52 (40)	
*ET (ms)*	***FGR***	103	162.76 ± 15.4	132–204 (164)	0.037*
	***Control***	114	167.05 ± 12.48	136–192 (168)	
*Aortic PSV (cm/s)*	***FGR***	103	67.59 ± 15.53	38.3–111 (66.1)	0.001**
	***Control***	114	75.65 ± 14.05	45.3–91.2 (75.9)	
*Pulmonary PSV(cm/s)*	***FGR***	103	58.58 ± 12.75	33-96.8 (57.4)	0.001**
	***Control***	114	65.74 ± 10.53	31.3–77.3 (70.2)	
*MPI*	***FGR***	103	0.51 ± 0.07	0.36–0.7 (0.51)	0.036*
	***Control***	114	0.5 ± 0.07	0.41–0.73 (0.49)	
*E wave (cm/s)*	***FGR***	103	29.5 ± 6.04	20.1–48.7 (28.9)	0.001**
	***Control***	114	32.84 ± 5.87	25.1–44.9 (32)	
*A wave (cm/s)*	***FGR***	103	38.69 ± 6.47	26.1–53.4 (38.3)	0.001**
	***Control***	114	44.23 ± 5.75	36.9–54.3 (43.2)	
*E/A Ratio*	***FGR***	103	0.77 ± 0.12	0.56–1.4 (0.75)	0.250
	***Control***	114	0.74 ± 0.09	0.56–0.89 (0.73)	
*FHR (bpm)*	***FGR***	103	143 ± 9.09	123–164 (143)	0.176
	***Control***	114	144.63 ± 8.15	130–161 (145)	
*Umbilical PI*	***FGR***	103	1.48 ± 0.55	0.65–2.53 (1.3)	0.001**
	***Control***	114	1.01 ± 0.14	0.79–1.21 (1)	
*Middle Cerebral Artery PI*	***FGR***	103	1.64 ± 0.43	0.79–2.68 (1.6)	0.011*
	***Control***	114	1.76 ± 0.28	1.33–2.35 (1.7)	
*Uterine-R PI*	***FGR***	103	1.34 ± 0.64	0.5–3.23 (1.18)	0.001**
	***Control***	114	0.86 ± 0.31	0.57–1.7 (0.75)	
*Uterine-L PI*	***FGR***	103	1.51 ± 0.72	0.5–3.8 (1.4)	0.001**
	***Control***	114	0.93 ± 0.23	0.6–1.5 (0.84)	
*Ductus Venosus PI*	***FGR***	103	0.59 ± 0.19	0.23–1.17 (0.56)	0.027*
	***Control***	114	0.53 ± 0.14	0.3–0.82 (0.5)	
*CPR*	***FGR***	103	1.32 ± 0.66	0.37–2.96 (1.17)	0.001**
	***Control***	114	1.76 ± 0.31	1.29–2.46 (1.79)	
*Apgar 1 st min*	***FGR***	103	6.25 ± 1.78	1–9 (7)	0.001**
	***Control***	114	7.16 ± 0.82	6–9 (7)	
*Apgar 5th min*	***FGR***	103	7.9 ± 1.23	3–10 (8)	0.063
	***Control***	114	8.26 ± 0.85	7–10 (8)	
*Birth Weight (g)*	***FGR***	103	1764.56 ± 686.54	440–3630 (1855)	0.001**
	***Control***	114	2353.16 ± 455.44	1855–3120 (2350)	

Kruskal‒Wallis test

*p < 0.05

**p < 0.01

 Fetuses in the FGR group were divided into two subgroups on the basis of the presence of absent end-diastolic flow in the umbilical artery: AEDF (+) and AEDF (–). These two subgroups, along with the control group consisting of normally growing fetuses, were compared in terms of demographic and echocardiographic parameters (Tables 2 and 3). The mode of delivery, amniotic fluid index (AFI), and neonatal intensive care unit (NICU) admission rates were evaluated among the groups.

**Table 2:** Comparison of mode of delivery, amniotic fluid index (AFI), and neonatal intensive care unit (NICU) admission rates among the AEDF (+), AEDF (–), and control groups

		Groups				*p*
*Mode of Delivery*		***AEDF +***	***AEDF -***	***Control***	***Total***	0,147
	***Primary Cesarean Section***	33 (%70.2)	26 (%46.4)	66 (%57.9)	125 (%57.6)	
	***Previous Cesarean Section***	10 (%21.3)	18 (%32.1)	24 (%21.1)	52 (%24)	
	***Induced Labor***	4 (%8.5)	12 (%21.4)	24 (%21.1)	40 (%18.4)	
*AFI*	***Normal***	38a (%80.9)	46a (%82.1)	114b (%100)	198 (%91.2)	***0***,001**
	***Oligohydramnios***	7a (%14.9)	10a (%17.9)	0b (%0)	17 (%7.8)	
	***Polyhydramnios***	2a (%4.3)	0a (%0)	0a (%0)	2 (%0.9)	
*NICU*	***No***	9a (%19.1)	32b (%57.1)	96c (%84.2)	137 (%63.1)	***0***,001**
	***Yes***	38a (%80.9)	24b (%42.9)	18c (%15.8)	80 (%36.9)	

Chi-square test **p < 0.01

**Table 3:** Comparison of measurements among the AEDF (+), AEDF (–), and control groups

		*n*	Ort ± Ss	Min–Max (Median)	*p*
*Age(years)*	***AEDF +***	47	29.83 ± 6.18	18–44 (30)	***0***,007**
	***AEDF -***	56	26.27 ± 5.41	18–43 (25.5)	
	***Control***	114	29.16 ± 6.47	19–44 (29)	
*Gravida*	***AEDF +***	47	2.11 ± 1.46	1–6 (1)	0.326
	***AEDF -***	56	1.96 ± 1.35	1–7 (1)	
	***Control***	114	2.37 ± 1.42	1–6 (2)	
*Parity*	***AEDF +***	47	0.77 ± 1.05	0–5 (0)	0.420
	***AEDF -***	56	0.64 ± 0.84	0–3 (0)	
	***Control***	114	0.95 ± 0.97	0–3 (1)	
*Abort*	***AEDF +***	47	0.3 ± 0.66	0–3 (0)	0.894
	***AEDF -***	56	0.34 ± 0.82	0–5 (0)	
	***Control***	114	0.37 ± 0.68	0–2 (0)	
*Gestatinal Age (weeks)*	***AEDF +***	47	28.85 ± 2.94	24–35 (28)	0.001**
	***AEDF -***	56	32.27 ± 3.49	23–36 (33)	
	***Control***	114	31.74 ± 3.02	27–36 (33)	
*Birth Week (week)*	***AEDF +***	47	32.15 ± 3.27	25–39 (33)	0.001**
	***AEDF -***	56	36 ± 2.32	26–41 (36.5)	
	***Control***	114	36.26 ± 2.02	34–41 (36)	
*Body Mass Index (kg/m2)*	***AEDF +***	47	28.98 ± 3.51	22.04–40.79 (28.16)	0.033*
	***AEDF -***	56	28.01 ± 3.64	21.05–39.3 (28.16)	
	***Control***	114	26.46 ± 3.11	21.91–33.5 (27.24)	
*ICT (ms)*	***AEDF +***	47	39.11 ± 7.45	24–60 (40)	0.484
	***AEDF -***	56	38.32 ± 7.8	24–60 (37)	
	***Control***	114	40.74 ± 7.92	24–64 (40)	
*IRT(ms)*	***AEDF +***	47	42.26 ± 6.12	32–60 (44)	0.041*
	***AEDF -***	56	45.79 ± 7.32	36–76 (44)	
	***Control***	114	42.11 ± 6.16	32–52 (40)	
*ET(ms)*	***AEDF +***	47	157.83 ± 15.42	132–188 (160)	0.001**
	***AEDF -***	56	166.89 ± 14.25	132–204 (168)	
	***Control***	114	167.05 ± 12.76	136–192 (168)	
*Aortic PSV (cm/s)*	***AEDF +***	47	63.62 ± 11.26	41.3–87 (64)	0.006**
	***AEDF -***	56	70.93 ± 17.79	38.3–111 (73.1)	
	***Control***	114	75.65 ± 14.37	45.3–91.2 (75.9)	
*Pulmonary PSV(cm/s)*	***AEDF +***	47	55.55 ± 11.91	37.6–96.8 (54)	0.001**
	***AEDF -***	56	61.12 ± 12.97	33-89.9 (59.25)	
	***Control***	114	65.74 ± 10.77	31.3–77.3 (70.2)	
*MPI*	***AEDF +***	47	0.52 ± 0.07	0.36–0.69 (0.5)	0.383
	***AEDF -***	56	0.51 ± 0.07	0.38–0.7 (0.51)	
	***Control***	114	0.5 ± 0.07	0.41–0.73 (0.49)	
*E wave (cm/s)*	***AEDF +***	47	27.25 ± 5.4	20.1–43.6 (25.6)	0.001**
	***AEDF -***	56	31.38 ± 5.94	21.4–48.7 (30.6)	
	***Control***	114	32.84 ± 6	25.1–44.9 (32)	
*A wave (cm/s)*	***AEDF +***	47	36.24 ± 6.42	26.1–52.2 (35.5)	0.001**
	***AEDF -***	56	40.75 ± 5.8	30.3–53.4 (39.95)	
	***Control***	114	44.23 ± 5.88	36.9–54.3 (43.2)	
*E/A Ratio*	***AEDF +***	47	0.75 ± 0.08	0.56–0.94 (0.75)	0.516
	***AEDF -***	56	0.78 ± 0.15	0.58–1.4 (0.75)	
	***Control***	114	0.74 ± 0.09	0.56–0.89 (0.73)	
*FHR (Bpm)*	***AEDF +***	47	144.49 ± 8.76	127–164 (146)	0.281
	***AEDF -***	56	141.75 ± 9.26	123–160 (141)	
	***Control***	114	144.63 ± 8.34	130–161 (145)	
*Umbilical PI*	***AEDF +***	47	2.03 ± 0.23	1.64–2.53 (2)	0.001**
	***AEDF -***	56	1.01 ± 0.19	0.65–1.49 (1.01)	
	***Control***	114	1.01 ± 0.14	0.79–1.21 (1)	
*Middle Cerebral Artery PI*	***AEDF +***	47	1.54 ± 0.45	0.79–2.68 (1.44)	0.001**
	***AEDF -***	56	1.72 ± 0.39	1.01–2.6 (1.73)	
	***Control***	114	1.76 ± 0.29	1.33–2.35 (1.7)	
*Right Uterine PI*	***AEDF +***	47	1.74 ± 0.57	0.74–2.86 (1.78)	0.001**
	***AEDF -***	56	1 ± 0.49	0.5–3.23 (0.87)	
	***Control***	114	0.86 ± 0.32	0.57–1.7 (0.75)	
*Left Uterine PI*	***AEDF +***	47	1.95 ± 0.71	0.85–3.8 (1.89)	0.001**
	***AEDF -***	56	1.15 ± 0.51	0.5–2.57 (0.95)	
	***Control***	114	0.93 ± 0.24	0.6–1.5 (0.84)	
*Ductus Venosus PI*	***AEDF +***	47	0.62 ± 0.22	0.23–1.17 (0.57)	0.290
	***AEDF -***	56	0.56 ± 0.16	0.3–0.99 (0.56)	
	***Control***	114	0.53 ± 0.15	0.3–0.82 (0.5)	
*CPR*	***AEDF +***	47	0.78 ± 0.26	0.37–1.42 (0.71)	0.001**
	***AEDF -***	56	1.77 ± 0.54	0.95–2.96 (1.69)	
	***Control***	114	1.76 ± 0.31	1.29–2.46 (1.79)	
*Apgar 1 st min*	***AEDF +***	47	5.38 ± 1.88	1–8 (6)	0.001**
	***AEDF -***	56	6.98 ± 1.31	2–9 (7)	
	***Control***	114	7.16 ± 0.83	6–9 (7)	
*Apgar 5th min*	***AEDF +***	47	7.32 ± 1.29	3–9 (7)	0.001**
	***AEDF (-)***	56	8.39 ± 0.95	6–10 (9)	
	***Control***	114	8.26 ± 0.87	7–10 (8)	
*Birth Weight (g)*	***AEDF +***	47	1321.91 ± 594.26	440–3000 (1230)	0.001**
	***AEDF -***	56	2136.07 ± 520.39	480–3630 (2070)	
	***Control***	114	2353.16 ± 465.86	1855–3120 (2350)	

Kruskal‒Wallis test

*p< 0.05

**p< 0.01

In terms of mode of delivery, the primary cesarean section rate was highest in the AEDF (+) group (70.2%), whereas it was 46.4% in the AEDF (–) group and 57.9% in the control group. The rate of cesarean deliveries following induction was notably low in the AEDF (+) group, at only 8.5%. However, these differences between the groups were not statistically significant (p = 0.147) (Table 2).

In pregnancies diagnosed with fetal growth restriction (FGR), when fetuses grouped according to umbilical artery Doppler findings were compared in terms of modified MPI values, the measurements were 0.52 ± 0.07 in the AEDF (+) group, 0.51 ± 0.07 in the AEDF (–) group, and 0.50 ± 0.07 in the control group (p = 0.383).

When other indicators of fetal cardiac function were analyzed, the ejection time (ET) was significantly shorter in the AEDF (+) group (157.8 ± 15.4 ms) than in the AEDF (–) group (166.9 ± 14.2 ms) and the control group (167.1 ± 12.8 ms) (p = 0.001). In the same group, the E- and A-wave peak velocities were also significantly lower (E wave: 27.25 ± 5.4 cm/s, p < 0.001; A wave: 36.24 ± 6.4 cm/s, p < 0.001).

As shown in Table 3, the E/A ratio was similar across groups (AEDF [+] 0.75 ± 0.08, AEDF [–] 0.78 ± 0.15, control 0.74 ± 0.09; p = 0.516), despite concurrent reductions in both E and A velocities in the AEDF (+) group. This pattern suggests declines in both early and late diastolic filling, accompanied by shortened systolic ejection time, reflecting early diastolic dysfunction in severe placental insufficiency.

 Spearman correlation analysis revealed that the modified left ventricular myocardial performance index (Mod-MPI) was significantly correlated with various cardiac and Doppler parameters across all groups. In the control group, a strong positive correlation was observed between Mod-MPI and the isovolumetric relaxation time (IRT) (r = 0.678, *p* < 0.001), whereas a negative correlation was found with the ejection time (ET) (r = –0.460, *p* < 0.001). Additionally, the Mod-MPI was positively correlated with gestational age (r = 0.473, *p* < 0.001), gravida (r = 0.450, *p* < 0.001), and the umbilical artery PI (r = 0.392, *p* < 0.001) and negatively correlated with the A wave PSV (r = –0.459, *p* < 0.001) and the ductus venosus PI (r = –0.265, *p* = 0.004).

 In the AEDF (+) group, Mod-MPI was significantly positively correlated with IRT (r = 0.602, p < 0.001) and isovolumetric contraction time (ICT) (r = 0.320, p = 0.028) and significantly negatively correlated with ET (r = –0.547, p < 0.001), the middle cerebral artery PI (MCA-PI) (r = –0.387, p = 0.007), and the cerebroplacental ratio (CPR) (r = –0.416, p = 0.004). A weak positive correlation was observed with the umbilical artery PI; however, this relationship was at the borderline of statistical significance (r = 0.251, p = 0.089).

 In the AEDF (–) group, the Mod-MPI showed a significant positive correlation with IRT (r = 0.459, p < 0.001) and ICT (r = 0.578, p < 0.001) and a significant negative correlation with ET (r = –0.506, p < 0.001) and A wave PSV (r = –0.307, p = 0.021). In this group, the correlations between Mod-MPI and the umbilical artery PI, MCA PI, and CPR were not statistically significant.

 ROC curve analyses for ET, E/A ratio, and Mod-MPI in relation to NICU admission and low 5-minute Apgar scores consistently yielded AUC values below 0.50, confirming their limited predictive utility when used in isolation. Although each parameter reflects important aspects of fetal cardiac and hemodynamic compromise, none served as a reliable solitary marker for adverse outcomes in this cohort. Their true clinical value is likely to emerge only when integrated into comprehensive multiparametric models alongside established Doppler indices such as UA PI and CPR.

**Fig. 2:**
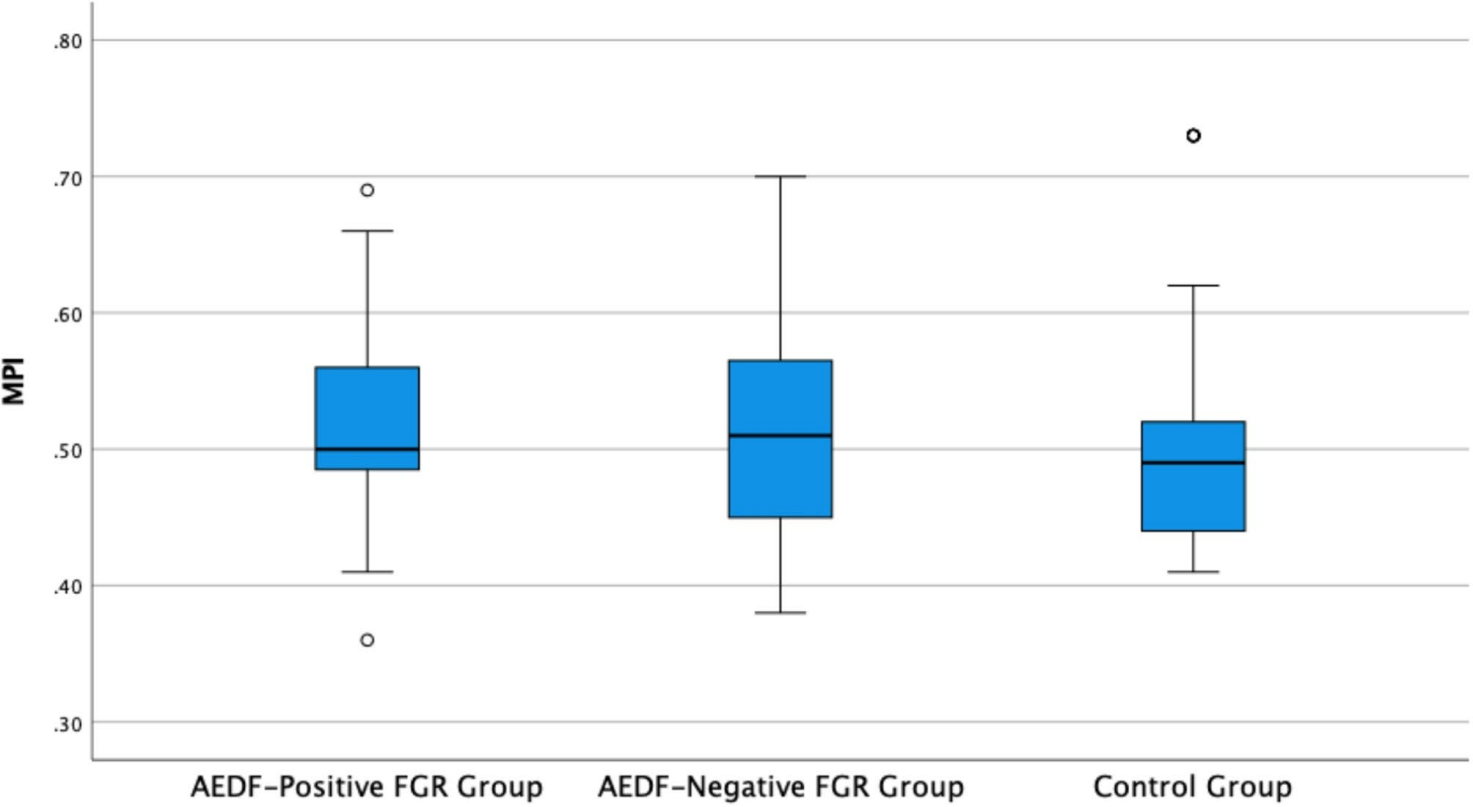
Box Plot of MPI by Group

 The comparison of MPI values among the study groups is illustrated in Fig. 1. The AEDF-positive FGR group demonstrated higher MPI values compared to both AEDF-negative FGR and control groups, indicating a trend toward worsening global cardiac function in cases with more severe placental insufficiency. In addition, the correlation matrix shown in Fig. 2 reveals the interrelationships between MPI and key Doppler parameters such as umbilical artery PI, ductus venosus PI, cerebroplacental ratio, as well as perinatal outcomes including 5-minute Apgar scores. These findings support the role of MPI as a comprehensive cardiac marker reflecting both hemodynamic compromise and its clinical implications in fetuses with growth restriction.

## Discussion

In this prospective study, the relationships among the modified left ventricular myocardial performance index (Mod-MPI), fetal cardiac timing parameters, and perinatal outcomes were evaluated in fetuses diagnosed with fetal growth restriction (FGR) grouped according to the presence or absence of absent end-diastolic flow (AEDF) in the umbilical artery, as well as in a control group. The findings revealed that in the AEDF (+) group, isovolumetric phases were significantly prolonged, the ejection time was shortened, and both the E- and A-wave velocities were decreased, resulting in an overall increase in Mod-MPI values. The significant reduction in both E- and A-wave velocities, particularly in combination with prolonged isovolumetric phases and elevated Mod-MPI values, indicates impaired diastolic function of the fetal left ventricle. This finding suggests dysfunction in both the passive and active phases of fetal cardiac filling. This group was also characterized by elevated umbilical artery PI, a reduced cerebroplacental ratio (CPR), lower Apgar scores, and increased NICU admission rates. Although the difference in MPI between the groups did not reach statistical significance, the strong correlations observed between MPI and timing parameters such as IRT, ICT, and ET suggest that the Mod-MPI may reflect the fetal heart’s pathophysiological response at an early stage.

 Fetal cardiac functions can be influenced not only by maternal factors such as hypertension, diabetes, and infections but also by fetal hemodynamic and structural conditions. Among these, intrauterine growth restriction (FGR) is considered one of the most significant conditions leading to impairment of the fetal cardiovascular system. In fetuses with FGR, the primary cause of ventricular remodeling is increased afterload resulting from elevated placental vascular resistance. However, increased afterload during fetal life is not unique to FGR; similar effects may also be observed in other conditions, such as in the recipient twin of twin-to-twin transfusion syndrome (TTTS), where hypervolemia leads to comparable hemodynamic stress. In such cases, the increased volume load and concomitant placental resistance can result in significant deterioration of the myocardial performance index (MPI). Taken together, these findings suggest that both increased placental vascular resistance and hypervolaemia can significantly affect fetal cardiac function [11].

 It has been demonstrated that as the severity of fetal growth restriction (FGR) increases—particularly in cases of early-onset and severe FGR—MPI values also increase in parallel, reflecting prenatal cardiac dysfunction [12]. In particular, it has been suggested that in FGR cases with abnormal umbilical artery Doppler findings (such as absent end-diastolic flow), left heart MPI values may be greater than those in FGR fetuses with normal Doppler flow. However, the findings in the literature remain inconsistent; some researchers have reported no significant difference in MPI even in the presence of AEDF and have suggested that the clinical utility of MPI is still not clearly established [13]. In our study involving 217 pregnancies, we evaluated the relationships among the left ventricular myocardial performance index (Mod-MPI), fetal cardiac timing parameters, and perinatal outcomes.

 In FGR cases resulting from uteroplacental insufficiency, the fetal cardiovascular system attempts to adapt to this chronic stress through compensatory mechanisms. Increased placental bed resistance leads to elevated afterload and reduced oxygen supply, causing subclinical changes that primarily affect diastolic function in the fetal heart. Prolonged hypoxic exposure impairs myocardial relaxation capacity and paves the way for the development of diastolic dysfunction. This condition may manifest early as reduced longitudinal myocardial motion and a delayed filling process. The redistribution of fetal circulation (brain-sparing mechanism) prioritizes flow to vital organs, while the heart working under increased hemodynamic load necessitates a comprehensive assessment of global cardiac function [14]. In our study, the FGR group presented significantly prolonged isovolumetric phase durations, shortened ejection times, and markedly reduced E- and A-wave velocities. These findings indicate that under chronic hypoxia, the fetal myocardium develops early diastolic dysfunction, which can be objectively assessed through the components of the modified MPI.

In the study by Bhorat et al., which evaluated 43 fetuses with FGR between 28 and 34 weeks of gestation, the left myocardial performance index (MPI) values measured by spectral Doppler significantly increased in parallel with the worsening of Doppler parameters. In cases of early-onset FGR, the mean MPI threshold was 0.59, whereas in the control group consisting of appropriate-for-gestational-age (AGA) fetuses, the value was 0.37. An MPI threshold of 0.54 provided 87% sensitivity and 75% specificity for predicting adverse perinatal outcomes, whereas a threshold of 0.67 was reported to yield 100% sensitivity and 81% specificity in predicting perinatal mortality. These findings suggest that MPI may serve as a potential prognostic marker reflecting fetal cardiac load and increased perinatal risk [15]. Similarly, in our present study, the mean MPI value was calculated as 0.51 ± 0.07 (median: 0.51; min– max: 0.36–0.70) in the 103 fetuses constituting the FGR group. In the control group, consisting of 114 fetuses with normal growth appropriate for gestational age, the mean MPI value was 0.50 ± 0.07 (median: 0.49; min–max: 0.41–0.73). The difference between the two groups was statistically significant (p = 0.036). These results suggest that the modified MPI may be a sensitive and clinically applicable cardiac marker for detecting subclinical myocardial dysfunction in FGR-induced cases.

While the MCA-PI is used to assess fetal cerebral perfusion, the cerebroplacental ratio (CPR), calculated as the MCA-PI/UA-PI ratio, is utilized to predict the risk of fetal hypoxia. Low CPR values have been associated with adverse perinatal outcomes such as emergency delivery and low Apgar scores. Although the prognostic power of the MCA-PI alone is limited, the DV-PI reflects the risk of intrauterine death, particularly in early FGR; however, its predictive value is diminished in late FGR [16]. In our study, the CPR values were significantly lower in the FGR group than in the control group. The reduction in CPR indicates the development of redistribution in fetal circulation toward the cerebral region, reflecting the activation of the classical “brain-sparing” adaptive response. Although this physiological mechanism aims to preserve cerebral perfusion in response to a reduced oxygen supply, it imposes increased hemodynamic stress on other organ systems, particularly cardiac structures. Indeed, the significantly elevated MPI values observed in the same group represent the fetal heart’s response to this adaptive process. The increase in MPI, accompanied by prolonged ICT and IRT durations and shortened ET, suggests that the fetal myocardium is experiencing heightened diastolic pressure and is approaching its compensatory limit. Therefore, the presence of elevated MPI values in conjunction with low CPR indicates diminished fetal systemic reserve and cardiac tolerance; when these two parameters are evaluated together, they may enable early identification of the risk for adverse perinatal outcomes.

In our study, significantly lower 1- and 5-minute Apgar scores were observed in the FGR group than in the control group, particularly among patients with AEDF (+). This finding supports the notion that fetal cardiac performance influences postnatal adaptive capacity. Since an increase in MPI reflects impairment in both systolic and diastolic functions, elevated MPI values are associated with reduced postnatal cardiac reserve and inadequate circulatory response. Similarly, the literature reports that FGR fetuses with high prenatal MPI values are more likely to have lower Apgar scores and an increased need for resuscitation [15–17]. However, when interpreting this relationship, it is important to consider that the AEDF (+) group delivered at earlier gestational ages, and other prematurity-related factors may also influence the Apgar score. In this context, MPI may serve not only as an indicator of intrauterine cardiac stress but also as an indirect marker of early neonatal cardiac function. The negative association observed between MPI and the Apgar score suggests that the modified MPI could be a clinically useful adjunctive parameter for the early identification of adverse perinatal outcomes.

Although, theoretically, right ventricular MPI measurement is considered more physiologically appropriate owing to right ventricular dominance in the fetal period, the left ventricle is generally preferred in clinical practice owing to technical challenges. Assessment of the right ventricle requires two separate imaging planes through the pulmonary and tricuspid valves, making it time-consuming and technically difficult to perform. In contrast, since the aortic valve (AoV) and mitral valve (MV) can be visualized within the same plane in the left ventricle, Doppler measurements have become more practical and reproducible. Indeed, the literature shows that MPI values derived from the left ventricle are more frequently reported than those from the right ventricle. However, the clinical utility of left ventricular MPI in the fetal period remains controversial. This inconsistency largely stems from technical variations in MPI measurement. Factors such as the device characteristics, insonation angle, and sweep speed can directly influence the measurement results. For example, Hernandez-Andrade et al. performed modified MPI measurements using an insonation angle of 0–30° and a sweep speed of 15 cm/s, whereas other studies have used narrower angles and different sweep speeds, making cross-study comparisons difficult [10, 18]. In our study, modified MPI measurements were performed from the left ventricle using an insonation angle of 0–15° and a sweep speed of 5 cm/s, aligning calipers at the peak points of the AoV and MV clicks. This method aims to reduce subjective variability in caliper placement and enhance measurement reproducibility. Owing to this standardized technique, MPI values across different gestational ages could be reliably compared.

Amniotic fluid volume primarily depends on fetal kidney filtration and urine production. Fetal renal artery Doppler measurements reflect renal perfusion, and these parameters have been reported to be associated with amniotic fluid levels [19]. In our study, when the FGR group [AEDF (+), AEDF (–)] was compared with the control group, significant differences were observed not only in MPI values but also in perinatal parameters such as mode of delivery, amniotic fluid index (AFI), and NICU admission rates. In particular, the AEDF (+) group had a higher rate of primary cesarean section, lower AFI levels, and a significantly increased frequency of oligohydramnios. These findings suggest that fetal circulatory dysfunction is not limited to the placental level but also adversely affects physiological processes such as renal perfusion, urine production, and fetal breathing movements. The increase in oligohydramnios may indicate reduced fetal urine output due to decreased renal perfusion, leading to the development of hypovolemia.

 In studies investigating the relationship between polyhydramnios and Mod-MPI, it has been reported that, in the presence of isolated polyhydramnios, the Mod-MPI values are significantly greater than those in control groups, and this increase may be associated with adverse perinatal outcomes [20]. In our study, polyhydramnios were detected in 2 patients (4.3%) in the AEDF (+) FGR group, and the Mod-MPI values in these patients were notably above the group average. In both cases, no structural anomalies or infectious etiologies were identified apart from isolated polyhydramnios; however, respiratory distress and a need for neonatal intensive care developed during the neonatal period. These findings suggest that increased amniotic fluid volume may be associated with fetal cardiac loading and myocardial dysfunction. Consistent with the literature, Mod-MPI may serve as a useful indicator for evaluating fetal cardiac reserve in cases of isolated polyhydramnios. However, larger-scale studies are needed to strengthen this conclusion.

 Fetal circulation is a dynamic system that is highly dependent on the oxygen and nutrient supplies from the uteroplacental bed, as well as the capacity of the fetoplacental system to transport these resources effectively. Any disruption within this system is first reflected by an increase in vascular resistance, followed by changes in blood flow patterns [21]. In our study, when the PI values of the umbilical artery, middle cerebral artery, and bilateral uterine artery were compared among the AEDF (+), AEDF (–), and control groups, statistically significant differences were detected (*p* < 0.001). The mean umbilical artery PI (UA-PI) in the AEDF (+) group was 2.03 ± 0.23, which was markedly greater than that in the AEDF (–) and control groups (1.01 ± 0.19 and 1.01 ± 0.14, respectively). This finding indicates a significant increase in placental vascular resistance in AEDF (+) patients and, in line with MPI, suggests increased loading in the fetal circulation. Similarly, middle cerebral artery PI (MCA-PI) values were lower in the AEDF (+) group (1.54 ± 0.45), which is consistent with the “brain-sparing response” that reflects preferential blood flow to the brain. The reduction in MCA-PI demonstrates that, as a compensatory response to fetal hypoxia, the fetal cardiovascular system prioritizes perfusion to central organs. The right and left uterine artery PI values were also significantly greater in the AEDF (+) group (1.74 ± 0.57 and 1.95 ± 0.71, respectively), indicating maternal-origin uterine perfusion insufficiency. These findings reveal that both fetoplacental and uteroplacental circulation are impaired in the AEDF (+) group and that this disruption adversely affects fetal hemodynamics and cardiac function, thereby increasing perinatal risk. In conclusion, elevated UA-PI and uterine artery PI values along with decreased MCA-PI reflect the hemodynamic signs of fetal distress, whereas the concurrently elevated MPI values support the notion that this stress also mirrors the level of cardiac function.

 In our cohort, the E/A ratio—calculated as an additional index of left ventricular diastolic function—was similar across groups, despite significant reductions in both E and A velocities in the AEDF (+) subgroup. This pattern suggests proportional declines in early and late diastolic filling, accompanied by shortened systolic ejection time, rather than selective impairment of one diastolic phase. While the composite Mod-MPI did not differ significantly between groups, individual components such as ET and IRT, when interpreted alongside conventional Doppler indices (UA PI, MCA PI, CPR), may offer incremental value in identifying fetuses at risk for adverse perinatal outcomes. Although ROC analyses in our dataset yielded AUC values below 0.50 for these individual parameters, the concept of a combined predictive model remains clinically appealing. Future multicenter prospective studies could integrate UA Doppler abnormalities, CPR<1.0, and MPI component alterations (ET shortening, prolonged IRT, reduced E/A ratio) into a multivariate algorithm to establish clinically applicable cut-offs and improve antenatal surveillance strategies in FGR.

### Limitations

Among the strengths of our study are its prospective design and the fact that all Doppler measurements were performed in a single center by an experienced perinatologist via a standardized protocol. However, being a single-center study may limit the generalizability of the findings to other populations or institutions. Modified left ventricular MPI measurements were obtained via the pulsed-wave Doppler method, which can be affected by factors such as fetal position, breathing movements, variability in heart rate, and operator-dependent technical variables. Although all the measurements were performed by an experienced specialist, small deviations in the timing components of the MPI—namely, IRT, ICT, and ET—may lead to significant differences in the total index. Long-term neurodevelopmental and cardiac follow-up data were not available, which limits the ability to correlate prenatal Doppler findings with later functional outcomes.

## Conclusion

 This study demonstrated that the modified left ventricular MPI is a noninvasive, time-sensitive, and multifaceted parameter for assessing fetal cardiac dysfunction. Particularly in AEDF (+) FGR cases, the presence of elevated MPI values accompanied by abnormal Doppler findings (increased UA-PI and uterine artery PI, decreased MCA-PI and CPR), along with adverse perinatal outcomes (low Apgar scores, increased NICU requirements), suggests that MPI reflects the pathophysiological processes that may serve both as an indicator and a consequence of fetal hemodynamic stress.

 This multilayered relationship between MPI and fetal circulatory parameters offers a perspective beyond conventional Doppler assessment and allows for early identification of compromised fetal cardiac reserve. The integration of MPI-based approaches into clinical algorithms may support more accurate and individualized decision-making in both the timing of delivery and postnatal neonatal management. However, to better understand this potential, large-scale, multicenter, and advanced prospective studies including postnatal cardiac outcomes are warranted.

## Data Availability

The datasets generated and/or analyzed during the current study are available from the corresponding author on reasonable request.
